# Mitochondrial dysfunction in Alzheimer’s disease: a key frontier for future targeted therapies

**DOI:** 10.3389/fimmu.2024.1484373

**Published:** 2025-01-14

**Authors:** Shuguang Wang, Zuning Liao, Qiying Zhang, Xinyuan Han, Changqing Liu, Jin Wang

**Affiliations:** ^1^ College of Traditional Chinese Medicine, Shandong University of Traditional Chinese Medicine, Jinan, China; ^2^ Department of Neurology, Fourth People’s Hospital of Jinan, Jinan, China; ^3^ Department of Internal Medicine, Jinan Municipal Government Hospital, Jinan, China

**Keywords:** mitochondria, AD, dysfunction, mechanisms, therapeutic targets

## Abstract

Alzheimer’s disease (AD) is the most common neurodegenerative disorder, accounting for approximately 70% of dementia cases worldwide. Patients gradually exhibit cognitive decline, such as memory loss, aphasia, and changes in personality and behavior. Research has shown that mitochondrial dysfunction plays a critical role in the onset and progression of AD. Mitochondrial dysfunction primarily leads to increased oxidative stress, imbalances in mitochondrial dynamics, impaired mitophagy, and mitochondrial genome abnormalities. These mitochondrial abnormalities are closely associated with amyloid-beta and tau protein pathology, collectively accelerating the neurodegenerative process. This review summarizes the role of mitochondria in the development of AD, the latest research progress, and explores the potential of mitochondria-targeted therapeutic strategies for AD. Targeting mitochondria-related pathways may significantly improve the quality of life for AD patients in the future.

## 1 Introduction

AD, currently the most common progressive neurodegenerative disease, predominantly affects individuals over 60 years old. Its primary clinical manifestations include memory loss and cognitive impairment. As of 2020, approximately 40 million people worldwide are affected by AD, with the number of patients expected to double within five years (1, 2). According to the latest data from China in 2023, there are nearly 9 million AD patients in the country, and this number is projected to increase four to five times by 2050 (3). With the increasing global elderly population, the quality of life of AD patients and the societal impact are receiving greater attention. The pathological features of AD include elevated levels of amyloid-beta (Aβ) peptides and hyperphosphorylated tau proteins (p-tau) (4). Aβ forms plaques, while tau proteins form neurofibrillary tangles, both closely related to cell death and brain tissue loss (5). The gut-brain axis is a newly proposed concept in recent years, emphasizing the significant impact of gut microbiota changes on brain health. In AD, dysbiosis of the gut microbiota promotes the onset and progression of AD through various mechanisms (6). First, an imbalance in the gut microbiota directly leads to the excessive release of proinflammatory cytokines, such as TNF-α and IL-6, increasing intestinal permeability and causing the “leaky gut” phenomenon. The damaged gut barrier allows harmful substances, including endotoxins like lipopolysaccharides (LPS), to enter the bloodstream and cross the blood-brain barrier (BBB), triggering inflammation in the central nervous system. The insulin resistance associated with AD may also stem from these inflammatory responses (7). Dysbiosis of the gut microbiota can activate inflammatory pathways such as NF-κB, further disrupting the insulin signaling pathway. This resistance is closely linked to metabolic abnormalities in the brain, which are considered key features of AD pathology. Additionally, recent research suggests that AD may originate in the gut and then spread to the brain. In one study, researchers injected Aβ1-42 oligomers into the gastric wall of mice and observed that these amyloid proteins migrated from the gut to the brain within a year, indicating that the translocation of Aβ oligomers may be associated with the onset of AD and neuroinflammation. Another study found that exposing aged Fischer 344 rats to curli-producing *Escherichia coli* increased the production of α-synuclein in the gut. Over time, more severe aggregation was observed in the brains of these rats. Elevated levels of TLR2, IL-6, and TNF-α were also detected in their brains, suggesting that bacterial amyloid proteins may induce α-synuclein aggregation through the cross-seeding mechanism, stimulating microglia and astrocyte proliferation, thus exacerbating AD pathology (8). Furthermore, experiments using APP transgenic mice showed that changes in gut microbial strains can affect Aβ deposition. These mice exhibited a reduction in *Firmicutes* and an increase in *Bacteroides* in the gut. Compared to APP transgenic mice raised under normal conditions, those raised in germ-free environments showed significantly reduced Aβ pathology in the brain (9). This further supports the notion that gut microbiota may promote AD-related pathology by regulating immune responses.

Mitochondria are known as the “powerhouses” of the cell, with their primary function being the production of ATP through oxidative phosphorylation, providing energy for the cell. In addition, mitochondria play vital roles in cellular metabolism, lipid synthesis, calcium homeostasis, and signal transduction. As the main source of reactive oxygen species (ROS), mitochondria normally produce small amounts of ROS during oxidative phosphorylation, which act as signaling molecules to regulate cellular functions. However, when mitochondrial function is impaired, excessive ROS production can lead to oxidative stress, causing damage to cellular structures and functions. Furthermore, mitochondria are crucial in regulating apoptosis (programmed cell death). When a cell experiences damage or stress, the permeability of the mitochondrial outer membrane increases, leading to the release of pro-apoptotic factors such as cytochrome c, which initiates the apoptotic pathway. In this way, mitochondria play a key role in maintaining the balance between cell survival and death. Mitochondria play a critical role in the pathogenesis of AD. As the core of cellular energy metabolism, mitochondria are responsible for oxidative stress regulation and apoptosis (10). In AD patients, mitochondrial dysfunction manifests as abnormal energy metabolism, increased oxidative stress levels, imbalanced mitochondrial dynamics, abnormal mitophagy, and aging (11). These abnormal processes lead to the aberrant cleavage of amyloid precursor protein (APP), subsequently promoting the production of Aβ. Additionally, they may trigger abnormal tau protein phosphorylation through complex signaling pathways and induce a cascade of inflammatory responses, further exacerbating neuronal damage (12–14).

**Figure 1:**
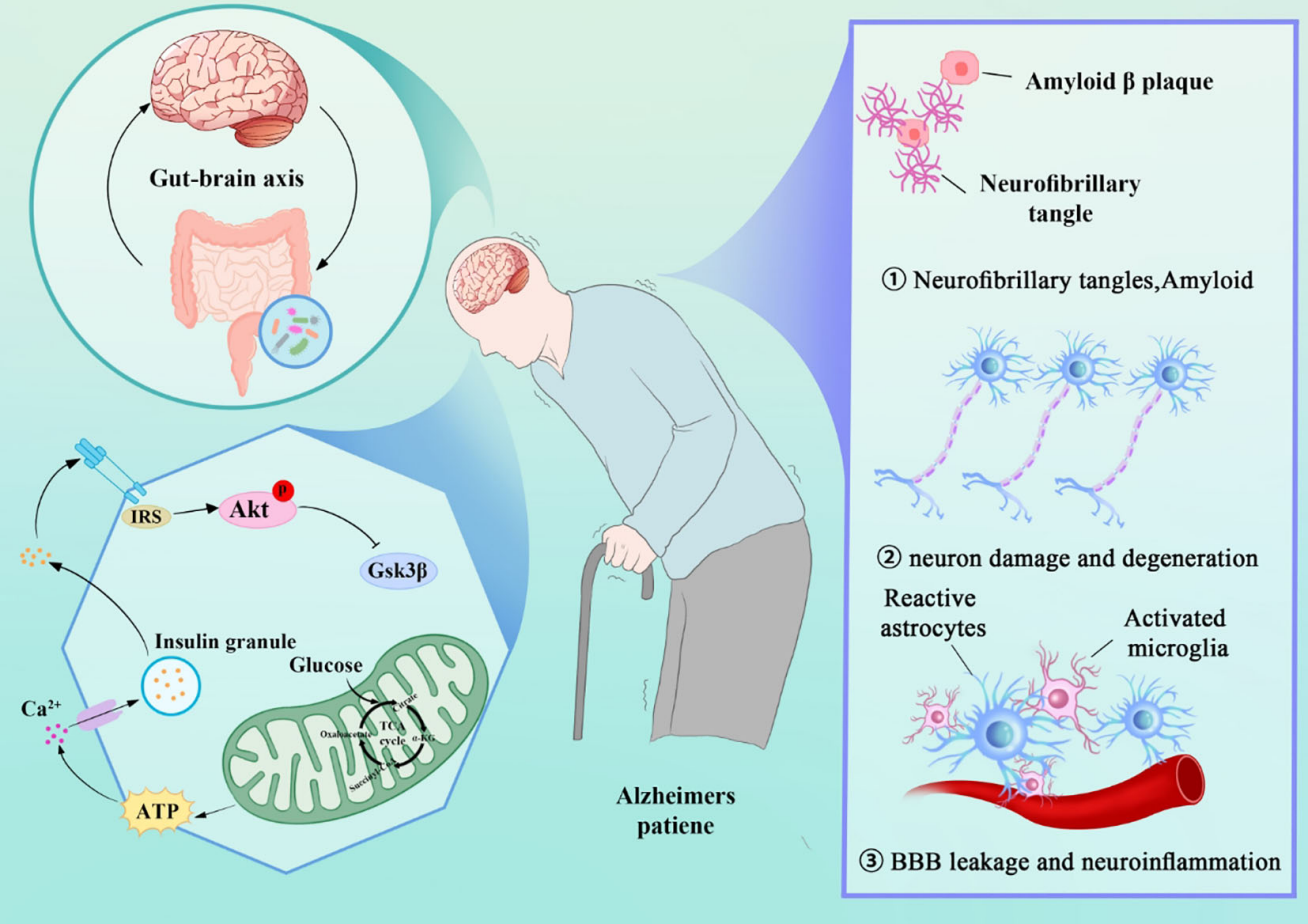
AD Mechanisms: Gut-Brain Axis, Mitochondrial Dysfunction, and Pathological Features (Tau, Amyloid, Neuroinflammation).

This paper aims to summarize the key roles of mitochondria in AD, exploring their specific mechanisms in the pathogenesis of AD. By gaining a deeper understanding of mitochondrial dysfunction in AD, this review provides a theoretical basis for the development of new targeted therapeutic strategies in the future. Here, we summarize the main mechanisms underlying the development and progression of AD (**Figure 1**).

## 2 Bioenergetic abnormalities in AD

Mitochondria serve as the central hub for energy production and metabolic regulation. In neuronal cells, oxidative phosphorylation (OXPHOS) occurs continuously on the inner mitochondrial membrane, leading to the release of a significant amount of ATP. Each molecule of glucose generates 36 molecules of ATP through OXPHOS. Essential nutrients, such as glucose and lipids, are converted into acetyl-CoA, which then enters the tricarboxylic acid cycle (TCA cycle), generating nicotinamide adenine dinucleotide (NADH) for ATP production via OXPHOS (15). During oxidative phosphorylation, substrates generated by the TCA cycle, such as NADH, actively transfer electrons to the electron transport chain (ETC), which is composed of four protein complexes (I-IV). The proton motive force (PMF) generated by electron transfer drives ATP synthase (Complex V) to synthesize ATP (16). In AD patients, mitochondrial dysfunction in the brain is particularly evident, leading to impaired energy metabolism. The activity of enzymes responsible for energy production, such as cytochrome c oxidase (COX), mitochondrial glutamate dehydrogenase (GDH), and α-ketoglutarate dehydrogenase (αKGDH), is significantly reduced (17, 18). In contrast, the activities of succinate dehydrogenase and malate dehydrogenase are significantly increased (19). Additionally, bioinformatics analyses have revealed significant abnormalities in mitochondrial-related metabolic pathways in AD patients, indicating defects in mitochondrial bioenergetics and reduced energy metabolism. Whole-genome transcriptomic studies of the posterior cingulate cortex show significantly reduced expression of nuclear genes encoding mitochondrial ETC subunits in AD (20). Moreover, microarray and RT-PCR studies indicate significant downregulation of key metabolic pathways such as the TCA cycle, glycolysis, and OXPHOS (21). Proteomic analyses further confirm these findings, showing that low expression of proteins in the OXPHOS pathway is one of the most severely affected events in the cortical brain of AD patients. Quantitative proteomics (LC-MS/MS-based iTRAQ) reveals specific alterations in mitochondrial proteins in AD brains, with significant dysregulation of mitochondrial complexes such as the respiratory chain complex and ATP synthase compared to age-matched controls. The post-translational modification processes of these enzymes are also critical, and their dysregulation leads to abnormal mitochondrial oxidative phosphorylation and accelerated permeability transitions of the mitochondrial membrane, both of which are tightly linked to AD occurrence (22). Additionally, pathological conditions like excessive Ca2+ transfer from the endoplasmic reticulum or increased cytosolic Ca2+ can impair mitochondrial metabolism, leading to oxidative stress, disrupted ion exchange, and reduced ATP production, all contributing to cell damage or even death (23). AD brains exhibit Ca2+ imbalance and mitochondrial dysfunction, reduced ATP levels, and increased reactive oxygen species (ROS) production, often present before the onset of AD symptoms (24). Bulk-RNA-seq data shows that seven genes exhibit consistent differential expression across various brain regions in AD patients, including three mitochondrial-encoded proteins: NDUFS5 (a subunit of mitochondrial complex I), SOD1 (superoxide dismutase 1), and OGT (O-GlcNAc transferase). The abnormal expression of these genes suggests mitochondrial dysfunction in AD, particularly in ATP production and antioxidant mechanisms. Additionally, the downregulation of NDUFS5 may affect ATP production, while the downregulation of SOD1 reduces the cell’s ability to combat oxidative stress.

## 3 Oxidative phosphorylation in AD

Mitochondria, as key regulators of cellular metabolism and energy, utilize NADH and FADH2 as energy carriers to drive ATP synthesis through the OXPHOS process mediated by the electron transport chain (ETC) (25). Mitochondria also serve as intracellular calcium reservoirs, maintaining cytosolic calcium homeostasis and participating in calcium signaling (26). In AD patients, mitochondria are significantly impacted by oxidative stress, with abnormal oxidative phosphorylation leading to elevated levels of reactive oxygen species (ROS) and accompanying calcium overload. These changes play a critical role in the onset and progression of AD (27). Excessive ROS can damage proteins, lipids, and DNA, triggering neuroinflammation. Given the high oxidative metabolic activity of neurons and their relatively weak antioxidant capacity, they are particularly sensitive to ROS (28). Additionally, ROS can disrupt the normal function of the ETC, particularly Complexes I and III. The resulting ETC dysfunction further increases ROS production, creating a vicious cycle that ultimately leads to neuronal apoptosis (29). Moreover, the excessive production of ROS can deactivate iron-sulfur clusters, causing a failure in electron transfer within the mitochondrial respiratory chain, further inhibiting mitochondrial energy production and accelerating the development of diseases such as AD. Mitochondrial oxidative stress is closely related to ferroptosis (30). The tricarboxylic acid (TCA) cycle in mitochondria regulates fatty acid metabolism and oxidative phosphorylation (OXPHOS), which are key processes in ferroptosis. Neuronal cells contain a substantial amount of mitochondrial ferritin, which regulates intracellular iron distribution, inhibits the Fenton reaction, and reduces ROS production and oxidative stress (31). Excessive ROS can also impair the normal function of calcium transport proteins and trigger the release of calcium from mitochondria, leading to a disruption of calcium homeostasis. The massive release of calcium ions from the mitochondria can alter the normal mitochondrial membrane potential, leading to the generation of more superoxide radicals. Once mitochondrial calcium overload occurs, it immediately triggers permeability transition, causing the outer mitochondrial membrane to rupture. Recent AD research increasingly focuses on the regulation of calcium homeostasis by the Calhm (calcium homeostasis modulator) family. Calhm1 controls calcium homeostasis and enhances neuronal resistance to Aβ toxicity, while Calhm2 regulates calcium ion influx and inflammatory activation in microglia. In mitochondrial dysfunction, abnormalities in Calhm1 and Calhm2 are observed, driving the progression of mitochondrial dysfunction (32). The accumulation of mitochondrial oxidative stress also leads to a gradual decline in the efficiency of mitochondrial energy production, converting more oxygen into ROS, further disrupting the defense system. In the context of aging, the accumulation of oxidative stress over time gradually undermines normal cellular defense mechanisms, leading to the progressive decline and functional impairment of tissues and organs (33). Oxidative stress causes cellular dysfunction, increased inflammation, and DNA mutations, thereby accelerating the aging process. With age, the expression levels of mitochondrial oxidase 3-nitrotyrosine significantly increase, leading to abnormal mitochondrial oxidative stress, further promoting mitochondrial dysfunction, and ultimately resulting in the onset of AD (34). Boosting antioxidant intake can mitigate ROS levels in the body. Mitochondrial ETC damage often leads to an increased NADH/NAD+ ratio. Normally, NADH transfers electrons to the ETC to regenerate NAD+, but when ETC activity is impaired, NADH accumulates in the cytoplasm. This accumulation hampers glycolysis and puts neuronal survival at risk. Furthermore, excess NADH can disrupt other metabolic processes, worsening the overall energy metabolism imbalance (35). Additionally, oxidative stress promotes inflammatory responses, playing a crucial role in the development and progression of AD. Excessive ROS activates classical inflammatory signaling pathways, promotes the production of pro-inflammatory factors, and triggers neuroinflammation. Neuroinflammation is central to AD-related pathological damage. The use of single-cell RNA sequencing (scRNA-seq) technology has further revealed cell type-specific gene expression changes in AD. Neurons (both excitatory and inhibitory) exhibit dysregulated mitochondrial gene expression in the early stages of AD pathology, particularly in pathways related to oxidative phosphorylation and mitochondrial transport. As the disease progresses, these dysregulated gene expressions become more pronounced in late-stage AD, reflecting the gradual loss of neuronal function. By integrating epigenomics, transcriptomics, and proteomics, researchers found that genes related to cellular respiration and oxidation were downregulated in AD patients, while genes associated with transcription and chromatin were upregulated. Additionally, histone modifications (such as H3K27ac and H3K9ac) were significantly increased in AD patients, suggesting that these epigenetic modifications may play a key role in the onset and progression of AD.

## 4 The role of mitochondrial dynamics imbalance in AD

Mitochondrial dynamics refer to the regulation of mitochondria within the cell through processes such as fusion, fission, movement, and autophagy. These processes are crucial for maintaining mitochondrial shape and function. Mitochondrial fusion helps integrate and optimize mitochondrial functions, especially in response to cellular metabolic stress, while mitochondrial fission allows the separation of damaged mitochondria for autophagy and clearance. Additionally, mitochondrial movement is vital for distributing energy to meet the cell’s needs. Together, these dynamic processes maintain cellular health and vitality. Mitochondria are not static organelles; instead, they are dynamic and capable of undergoing fission and fusion to adapt to various environmental changes (36). At any point in the cell cycle, mitochondria may divide into two independent mitochondria or undergo fusion by disrupting and reassembling their inner and outer membranes to form complete mitochondria. Recent studies have identified several proteins closely related to mitochondrial dynamics, including Drp1, mitofusins 1/2 (Mfn1/2), mitochondrial fission protein 1 (Fis1), and mitochondrial fission factor (Mff) (37, 38). The onset and progression of AD are closely associated with the disruption of mitochondrial dynamics balance, likely damaging the distribution and structure of mitochondria in neurons during the pathological stages of AD (39, 40). Detailed morphological measurements of AD-susceptible neurons have shown a significant reduction in mitochondrial numbers, accompanied by a notable increase in mitochondrial size. Confocal and electron microscopy analyses further confirm the presence of substantial mitochondrial fragmentation and morphological defects in AD patients (41).

In Drp1 and Mfn2 knockout mouse models, mitochondrial morphology in the brain exhibits similar fragmentation characteristics, indicating that uncontrolled mitochondrial fission leads to mitochondrial fragmentation (42). Significant changes in the expression of proteins related to mitochondrial fission and fusion in the brains of AD patients confirm the concept of mitochondrial dynamics imbalance in AD neurons (43). In AD, the expression of Fis1 and Drp1 increases, while the expression of Mfn1/2 and Opa1 decreases significantly (44). However, a few studies have reported downregulation of Drp1 expression, suggesting significant heterogeneity among AD patients (45). Mitochondrial fusion is largely controlled by three GTPase proteins: Mfn1 and Mfn2, located on the outer mitochondrial membrane (TOM), and Opa1, which resides on the inner mitochondrial membrane (TIM). Mfn1 and Mfn2 work in concert to mediate the fusion of the outer membrane, with their interaction being crucial for this process. In contrast, Opa1 primarily regulates the fusion of the inner mitochondrial membrane, ensuring the proper structure and dynamic equilibrium of the inner membrane. Mitochondrial fission, in contrast, occurs through two main pathways: one is when healthy mitochondria actively divide in the middle to form two halves due to insufficient mitochondrial numbers; the other is when damaged mitochondria divide from the ends, resulting in a large segment and a small segment, with the damaged small segment being actively eliminated (46). Excessive mitochondrial fission directly impairs the ETC, disrupts mitochondrial membrane potential, and increases ROS production, thereby exacerbating oxidative stress within mitochondria. Research has shown that Aβ closely interacts with Drp1, and this strong interaction directly leads to excessive ROS generation. Elevated levels of ROS, in turn, further activate the expression of Drp1 and Fis1, resulting in excessive mitochondrial fission and ultimately causing synaptic dysfunction. Additionally, studies have found that Drp1 interacts with phosphorylated tau protein, enhancing Drp1 activity and further exacerbating mitochondrial fission in AD patients (47, 48). Additionally, S-nitrosylation and phosphorylation of Drp1 are significantly increased in the brain tissue of AD patients, and S-nitrosylation of Drp1 enhances GTPase activity, thereby promoting mitochondrial fission. In AD models, Drp1 actively translocates from the cytoplasm to Fis1 on the TOM or binds to Mff, initiating the mitochondrial fission process (49).

## 5 Mitochondrial genome in AD

Mitochondrial DNA (mtDNA) is crucial for normal mitochondrial function, but due to its proximity to reactive oxygen species (ROS) production sites and a lack of effective DNA protection and repair mechanisms, it is susceptible to oxidative stress and damage, leading to mutations (50, 51). These mutations can result from genetic inheritance or gradually accumulated somatic mutations. Through clonal expansion, mtDNA mutations can spread, affecting mitochondrial function and ultimately compromising cell viability (52). Scientists utilized a knock-in mouse model with a proofreading-deficient mitochondrial DNA polymerase (PolgAD257A) to study this phenomenon (53). The results showed that as these mice aged, they accumulated mtDNA mutations, leading to premature aging and bioenergetic defects associated with mitochondria. This suggests that reduced mtDNA-encoded bioenergetic enzyme subunits may hinder mitochondrial enzyme production (54). Studies using AD cell models, where mtDNA-deficient cell lines are merged with enucleated cells from AD patients, have directly associated mtDNA changes with enzyme dysfunction. Furthermore, it has been observed that mtDNA damage occurs more frequently in individuals with mild cognitive impairment (MCI) and AD compared to control groups.This damage may result from age-related nucleotide damage or maternally inherited mutations. For instance, reduced COX activity in asymptomatic individuals suggests a possible hereditary component. Numerous studies have focused on somatic mtDNA mutations in AD, particularly the common 5-kb deletion (mtDNA 4977), which affects ETC complexes I, III, and V. This deletion has been found to increase 15-fold in the prefrontal cortex with age, especially in younger AD patients (55). A comprehensive analysis of mtDNA rearrangements shows a significant rise in F-type and R-type rearrangements in AD brains (56). Moreover, heteroplasmic mtDNA point mutations in the control region (CR) increase by an average of 63% in AD brains (57, 58). Research suggests that functional deficiencies in DNA repair enzyme activities in AD brain tissue may lead to this process’s absence. Reduced mtDNA copy number in AD brains correlates with poor base excision repair (BER) capacity (59). Further studies indicate that oxidative stress is the fundamental cause of age-dependent mtDNA damage. Compared to age-matched controls, oxidative damage to mtDNA in AD patients’ brains is tripled, and oxidative bases are almost tenfold higher than in nuclear DNA (60). Elevated levels of oxidative nucleic acids in the mtDNA of preclinical AD patients indicate that this could be an early marker of the disease. Recent studies have also reported reduced OGG1 activity and compromised BER capacity in both AD and MCI patients, suggesting that replication errors may significantly contribute to the rise in mtDNA mutations observed in AD (61, 62). Additional modifications to mtDNA could further affect its transcription and functionality. For instance, pathological samples from AD patients exhibit increased 5-methylcytosine levels in the mtDNA D-loop region, while late-onset AD patients show decreased methylation in the D-loop region of peripheral blood. The relevance of these findings to AD pathophysiology warrants further investigation (63). Variations, mutations, and modifications in mtDNA are likely to play critical roles in AD pathophysiology. The mitochondrial cascade hypothesis suggests that inherited mtDNA variations influence individual susceptibility, whereas the accumulation of brain somatic mtDNA variations and mutations, driven by environmental factors during aging, shapes phenotypic expression (64). However, specific mtDNA alterations and their potential causal roles in AD pathophysiology have yet to be established. Notably, mtDNA changes are also observed in other neurodegenerative diseases, but these changes are not specific to AD, and their specific relationship with AD-related changes requires further exploration.

## 6 Mitophagy in AD

**Figure 2:**
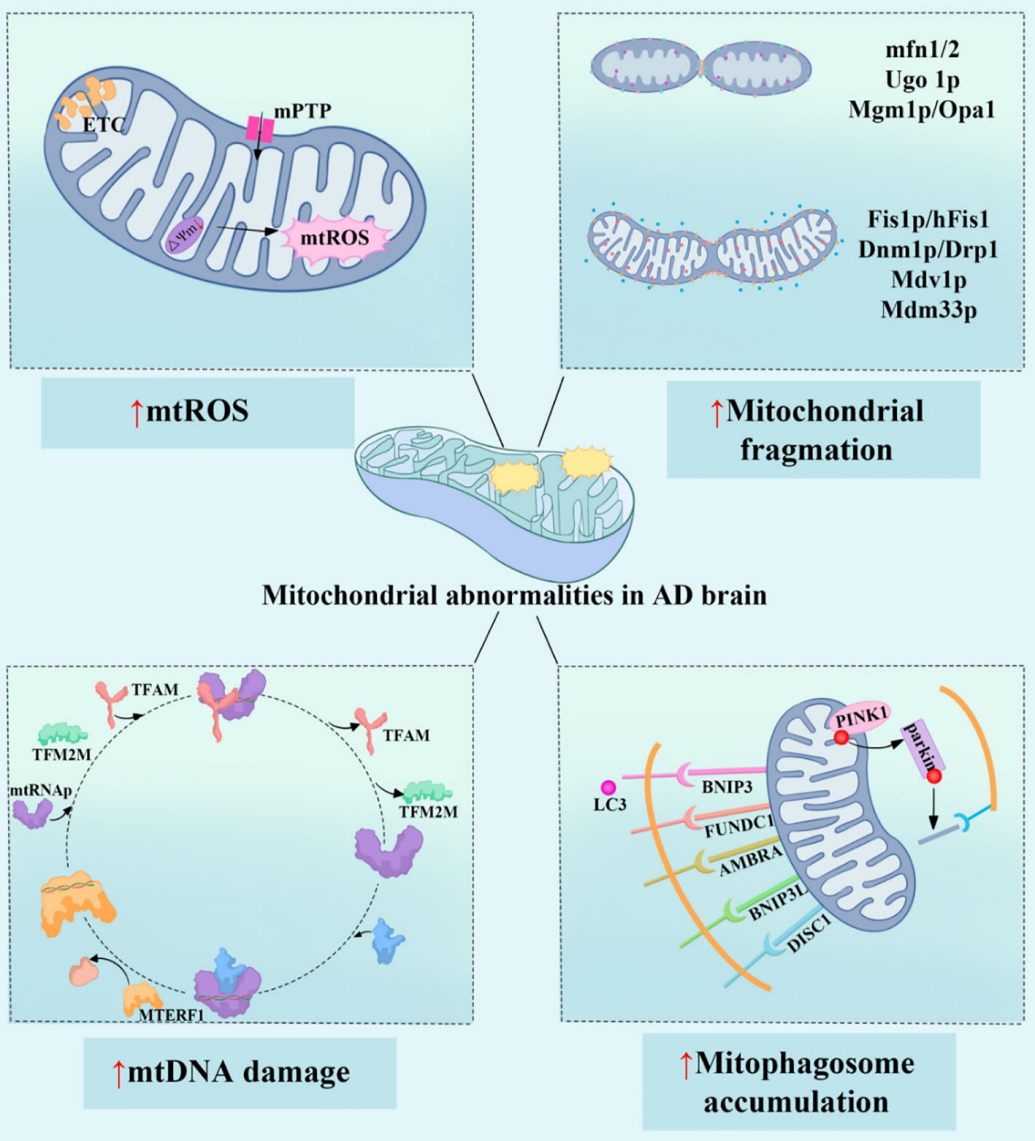
Mitochondrial dysfunction promotes the onset and progression of AD. The four main mechanisms of mitochondrial dysfunction in AD patients include: increased mtROS (mitochondrial reactive oxygen species), leading to oxidative stress and cellular damage; imbalance in mitochondrial dynamics, caused by dysregulation of mitochondrial fusion and fission proteins (such as mfn1/2 and Drp1), which exacerbates mitochondrial structural damage and functional impairment; mtDNA (mitochondrial DNA) damage, affecting mitochondrial gene expression and function, involving factors such as TFAM and MTERF1; and the abnormal accumulation of mitophagosomes, indicating impaired mitophagy, where damaged mitochondria are not cleared in time, with proteins such as PINK1 and BNIP3 being involved in this process. These mitochondrial abnormalities interact with each other, leading to metabolic dysfunction, cell apoptosis, and inflammatory responses, ultimately accelerating the neurodegenerative changes in AD. mtROS, Mitochondrial Reactive Oxygen Species; ETC, Electron Transport Chain; mPTP, Mitochondrial Permeability Transition Pore; mfn1/2, Mitofusin 1/2; Ugo1p, Ugo Protein 1; Mgm1p, Mitochondrial Genome Maintenance 1 Protein; Optic Atrophy Protein 1; Fis1p/hFis1, Fission 1 Protein/Human Fission 1; Dnm1p/Drp1, Dynamin-related Protein 1; Mdv1p, Mitochondrial Division Protein 1; Mdm33p, Mitochondrial Distribution and Morphology Protein 33; TFAM, Mitochondrial Transcription Factor A; TFB2M, mitochondrial transcription factors B2; MTERF1, Mitochondrial Transcription Termination Factor 1; BNIP3, Bcl-2/Adenovirus E1B 19 kDa Interacting Protein 3; FUNDC1, FUN14 Domain Containing Protein 1; AMBRA1, Activating Molecule in BECN1-Regulated Autophagy Protein 1; BNIP3L, BCL2/Adenovirus E1B 19 kDa Protein-Interacting Protein 3-Like; DISC1, Disrupted in Schizophrenia 1 Protein; PINK1, PTEN-Induced Kinase 1.

Mitophagy is a selective autophagy process that is triggered by factors such as mitochondrial damage or cellular aging. Following mitochondrial depolarization and damage, mitochondria are encapsulated by a double-membrane structure and subsequently degraded through lysosomal fusion (65, 66). Mitophagy removes damaged mitochondria, contributing to the maintenance of normal cellular function and the balance of mitochondrial quantity and quality. Currently, three known mechanisms regulate mitophagy: the PINK1/Parkin pathway, which modulates mitochondrial depolarization and facilitates the clearance of damaged mitochondria (67, 68). The BNIP3/NIX pathway, which is associated with erythrocyte maturation, where BNIP3 and NIX can bind to LC3 to directly mediate mitophagy (69). The FUNDC1-mediated autophagy pathway, where FUNDC1, a mitochondrial outer membrane receptor protein, binds to LC3 under hypoxic conditions to promote mitophagy (70). FUNDC1 can also interact with HSC70, recruiting misfolded proteins to the mitochondria through the TOM-TIM complex for degradation by the mitochondrial protease LONP1 (71). In AD patients, mitophagy dysfunction accelerates the accumulation of related mitochondrial autophagosomes in neurons, impairing synaptic function and structure, ultimately leading to cognitive deficits. Studies have shown that the PINK1/Parkin signaling pathway plays a predominant role in mediating mitophagy. In AD patients, the loss of Parkin protein directly promotes neuroinflammation (72). PINK1, another key protein in the Parkin signaling pathway, is primarily regulated by PTEN. Mitochondrial depolarization prevents the normal processing and degradation of PINK1, leading to its accumulation on the mitochondrial outer membrane (73). The accumulation of PINK1 recruits and activates Parkin. Typically, Parkin ubiquitinates intracellular Aβ, preventing its formation into extracellular plaques, thereby avoiding neurotoxicity. However, in AD patients, deubiquitinases such as USP30 and USP35 are overexpressed, enhancing Aβ stability and inducing neurotoxicity. AMPK and mTORC1 are key upstream kinases that initiate mitophagy (74). When cellular energy is depleted, AMPK is activated or mTORC1 is inhibited, leading to the phosphorylation of the ULK1 complex. The ULK1 complex activates PtdIns3K, recruiting PI3P effector proteins to form a pre-autophagosomal structure, thus initiating mitophagy (75). AD is a complex multifactorial disease where various pathological changes contribute to disease progression. Impaired mitophagy leads to the accumulation of abnormally modified proteins, such as Aβ and tau. These abnormal proteins interact, promoting the onset and progression of AD (76). For instance, tau inhibits the transport of APP to axons and dendrites, causing its accumulation in the cell body. Numerous studies have confirmed that Aβ and tau disrupt the normal process of mitophagy and have a combined or synergistic effect (77). Different pathological tau proteins can exacerbate Aβ-induced mitochondrial damage. Caspase-3 actively cleaves tau at Asp421 before the formation of neurofibrillary tangles, resulting in the release of large amounts of tau, thereby promoting the early onset of AD. Pathological tau in primary neurons can exacerbate mitochondrial localization, transport abnormalities, and oxidative stress induced by low concentrations of Aβ. In mature neurons, overexpression of pseudophosphorylated PHF-1 site (S396/S404) tau does not significantly alter mitochondrial morphology and normal function (78). However, Aβ causes mitochondrial membrane potential loss and increases SOD levels. Although overexpression of APP and tau enhances autophagic flux, the recruitment of Parkin and PINK1 to mitochondria is significantly reduced, leading to mitophagy abnormalities (79). Combined overexpression of APP and tau results in the accumulation of depolarized mitochondria. Researchers observed that in mouse models, many proteins were significantly dysregulated, with one-third being mitochondrial proteins, primarily associated with OXPHOS complexes I and IV (80, 81). Dysregulation of complex IV activity is related to Aβ, while dysregulation of complex I is associated with tau. Thus, mitochondrial dysfunction appears early in AD, but subsequent Aβ accumulation and tau pathology, either individually or synergistically, exacerbate mitochondrial damage. This leads to the accumulation of damaged and dysfunctional mitochondria, closely linked to impaired mitophagy. Above, we have thoroughly summarized how mitochondrial dysfunction contributes to the onset and progression of AD by promoting abnormal metabolism, oxidative stress, mitochondrial dynamics imbalance, and impaired mitophagy (**Figure 2**).

## 7 Targeted therapeutic strategies

Mitochondrial dysfunction is a key factor in AD, offering potential therapeutic targets, whether as an initial or subsequent event. However, the double-membrane structure of mitochondria poses a significant challenge for drug delivery. While TOM allows passage of molecules with medium to low molecular weight, TIM is even less permeable, further complicating treatment strategies (82). Therefore, exploring efficient mitochondrial-targeted drug delivery strategies for precise medication and regulation in AD treatment is of great significance (**Table 1**).

**Table 1:** Therapeutic Use of mitochondria-related drugs in AD.

Category	Drug	Mechanism	Ref
Mitochondrial Energy Metabolism	Alpha-lipoic acid, MitoQ	Antioxidant, reduces ROS production, protects mitochondria	(37)
Mitochondrial Energy Metabolism	Coenzyme Q10	Enhances electron transport chain, improves ATP production	(97)
Mitochondrial Energy Metabolism	Rhodiola rosea extract	Reduces oxidative stress, protects mitochondrial membrane	(98)
Mitochondrial Dynamics	Mdivi-1	Inhibits Drp1, prevents excessive fission	(99)
Mitochondrial Dynamics	Dynasore	Dynamically regulates mitochondrial fusion and fission	(100)
Mitochondrial Quality Control	Rapamycin	Inhibits mTOR pathway, enhances autophagy	(101)
Mitochondrial Quality Control	EGCG (Epigallocatechin gallate)	Activates AMPK, maintains mitochondrial homeostasis	(102)
Mitochondrial Quality Control	PQQ (Pyrroloquinoline quinone)	Promotes mitochondrial biogenesis, enhances metabolism	(103)
Mitophagy Restoration	Resveratrol	Activates Sirt1-Parkin pathway, enhances mitophagy	(104)
Mitophagy Restoration	Metformin	Upregulates Nrf2 signaling, promotes mitochondrial autophagy	(105)
Mitophagy Restoration	Spermidine	Induces autophagy, reduces mitochondrial damage	(106)

### 7.1 Strategies to regulate mitochondrial activity

Targeting mitochondrial activity to directly enhance bioenergetics is a crucial strategy in AD treatment. Oxaloacetate (OAA), an intermediate compound in the TCA cycle and gluconeogenesis, has been shown to enhance overall brain energy status by increasing the efficiency of energy metabolism and upregulating gene expression or enzyme activity related to mitochondrial function (83). Currently, OAA is undergoing Phase I clinical trials to assess its potential efficacy in AD treatment. Recent studies have identified that the compound CP2 can slightly inhibit the FMN subunit (NDUFA1) of Complex I, which helps enhance mitochondrial respiratory capacity and reduce proton leakage (84). CP2 has demonstrated the ability to alleviate cognitive impairment and pathological features in various AD animal models and is being developed as a potential therapeutic for AD (85). However, another study indicated that neuronal ablation of NDUFA5, another subunit of Complex I, resulted in mild chronic encephalopathy in mice, suggesting the need for extreme caution in selecting bioenergetic modulators (86). Without sufficient safety, these drugs may lead to serious side effects, and finding a bioenergetic modulator that is both effective and safe for AD treatment remains a critical challenge.

### 7.2 Restoration of mitochondrial integrity

In AD, the loss of mitochondrial structural and functional integrity may be responsible for energy metabolism disorders and increased oxidative stress. Therefore, developing therapeutic strategies aimed at restoring mitochondrial integrity holds significant potential (87). Achieving this goal requires a deep understanding of the relevant mitochondrial mechanisms, including mitochondrial dynamics, biogenesis, and mitophagy, as well as the interactions between mitochondria and other organelles (88). MitoQ, a mitochondria-targeted antioxidant, can penetrate directly into mitochondria and help neutralize free radicals, thereby reducing oxidative stress. Although MitoQ has shown potential therapeutic effects in animal models, further clinical trials are needed to validate its application in AD patients (89, 90). SS-31, a small peptide capable of penetrating the mitochondrial membrane, exhibits both antioxidant and anti-apoptotic properties. By stabilizing the mitochondrial membrane and reducing oxidative stress, SS-31 protects mitochondrial function (91). In preclinical studies of AD, SS-31 has shown potential to improve mitochondrial function and cognitive abilities, and it is currently undergoing related clinical trials.

### 7.3 Application of nanoparticles in mitochondrial targeting

Nanoparticles, due to their adjustable particle size and modifiable surface, can significantly enhance targeting efficiency and increase drug bioavailability, making them an effective approach for the treatment of AD (92). Since the mitochondrial membrane is rich in cardiolipin, researchers have developed liposome- and polymer-based nanoparticles to more effectively target mitochondria. To harness the strong negative potential of TIM, researchers have designed cationic compounds such as DQA and TPP derivatives for targeted mitochondrial delivery (93). The FDA-approved cationic lipid DQA has been used to create DQAsomes, which are liposome-like structures that interact with plasmid DNA (pDNA) through electrostatic forces. However, the gene transfection efficiency of DQAsomes in mitochondria is still relatively low (94). To address this issue, researchers have combined DQA with other compounds to enhance cellular uptake and endosomal escape, thereby improving gene expression within mitochondria (95). Additionally, the lipophilic cation TPP can enhance the electrostatic interaction between delivery systems and mitochondria. For example, nanoparticles formed by coupling TPP with polyethylene glycol-polyethyleneimine (PEG-PEI) through amide bonds have significantly improved the uptake and internalization of pDNA within mitochondria (96). We summarize clinical trials targeting mitochondrial targets in AD (**Table 2**).

**Table 2:** Clinical trials targeting mitochondrial targets in AD.

NCT Number	Study Title	Study Status	Conditions	Interventions	Phases
NCT05617508	N-DOSE AD: A Dose Optimization Trial of Nicotinamide Riboside in Alzheimer’s Disease	RECRUITING	Alzheimer Disease|Dementia	OTHER: Placebo|DIETARY_SUPPLEMENT: Nicotinamide Riboside supplementation 1000mg daily in total|DIETARY_SUPPLEMENT: Nicotinamide Riboside dose escalation (up to 3000 mg daily in total)	PHASE2
NCT04430517	Effects of Nicotinamide Riboside on Bioenergetics and Oxidative Stress in Mild Cognitive Impairment/Alzheimer’s Dementia	RECRUITING	Mild Cognitive Impairment|Mild Alzheimer Disease	DRUG: Nicotinamide riboside	EARLY_PHASE1
NCT03978052	Prevention of Cognitive Decline in ApoE4 Carriers With Subjective Cognitive Decline After EGCG and a Multimodal Intervention	COMPLETED	Alzheimer Disease|Cognitive Decline	DIETARY_SUPPLEMENT: EGCG|DIETARY_SUPPLEMENT: Placebo EGCG|OTHER: Healthy lifestyle recommendations|OTHER: Multimodal lifestyle intervention	nan
NCT03630419	Specialized Food Plan Based on Individual Physiological Comprehensive Body Assessments Accompanied With Cellular Repair Therapy to Decrease Inflammation Cognitively Impaired Patients	COMPLETED	Mild Cognitive Impairment|Alzheimer Disease	DIETARY_SUPPLEMENT: Mito-Food Plan|OTHER: Cellular Repair Therapy	PHASE1|PHASE2
NCT03101085	S-Equol in Alzheimer’s Disease 2 Trial	COMPLETED	Alzheimer Disease	DRUG: S-equol and Placebo|DRUG: Placebo and S-equol	PHASE1|PHASE2
NCT02142777	S-Equol in Alzheimer’s Disease (SEAD) Trial	COMPLETED	Alzheimer’s Disease	DRUG: S -Equol|DRUG: Placebo	PHASE1
NCT00951834	Sunphenon EGCg (Epigallocatechin-Gallate) in the Early Stage of Alzheimer´s Disease	COMPLETED	Alzheimer’s Disease	DRUG: Epigallocatechin-Gallate|DRUG: Placebo	PHASE2|PHASE3
NCT00939783	An Extension To The B1451027 Protocol To Evaluate The Long Term Safety And Tolerability Of Dimebon In Patients With Alzheimer’s Disease	TERMINATED	Alzheimer’s Disease	DRUG: Dimebon	PHASE3
NCT00838110	A Phase 3 Study To Evaluate The Safety And Tolerability Of Dimebon Patients With Mild To Moderate Alzheimer’s Disease	COMPLETED	Alzheimer’s Disease	DRUG: Dimebon|DRUG: Placebo|DRUG: Dimebon|DRUG: Placebo	PHASE3
NCT00829374	Safety and Efficacy Study Evaluating Dimebon in Patients With Mild to Moderate Alzheimer’s Disease on Donepezil	COMPLETED	Alzheimer’s Disease	DRUG: Dimebon|DRUG: Dimebon|DRUG: Placebo comparator	PHASE3
NCT00825084	A Phase 1 Study To Evaluate The Pharmacokinetics, Safety, And Tolerability Of Dimebon [PF-01913539] In Japanese And Western Healthy Subjects	COMPLETED	Alzheimer’s Disease|Huntington’s Disease	DRUG: Dimebon|DRUG: Dimebon|DRUG: Dimebon|DRUG: Dimebon	PHASE1
NCT00704782	Study of Combination Therapy With Dimebon and Donepezil (Aricept) in Patients With Alzheimer’s Disease	TERMINATED	Alzheimer’s Disease	DRUG: dimebon	PHASE2
NCT00675623	A Safety and Efficacy Study of Oral Dimebon in Patients With Mild-To-Moderate Alzheimer’s Disease	COMPLETED	Alzheimer’s Disease	DRUG: Dimebon|DRUG: Dimebon|DRUG: Placebo	PHASE3
NCT05617508	N-DOSE AD: A Dose Optimization Trial of Nicotinamide Riboside in Alzheimer’s Disease	RECRUITING	Alzheimer Disease|Dementia	OTHER: Placebo|DIETARY_SUPPLEMENT: Nicotinamide Riboside supplementation 1000mg daily in total|DIETARY_SUPPLEMENT: Nicotinamide Riboside dose escalation (up to 3000 mg daily in total)	PHASE2
NCT03514875	Effects of Mitochondrial-targeted Antioxidant on Mild Cognitive Impairment (MCI) Patients	WITHDRAWN	Alzheimer Disease, Early Onset|Mild Cognitive Impairment	DIETARY_SUPPLEMENT: MitoQ|DIETARY_SUPPLEMENT: Placebo	NAN

## 8 Conclusion

The mechanisms by which mitochondrial dysfunction contributes to the onset and progression of AD are gradually being elucidated. Mitochondria in AD patients often exhibit significant morphological abnormalities, specifically manifesting as mitochondrial swelling, cristae distortion, and an abnormal increase in mitochondrial size. These morphological changes are typically caused by alterations in the expression levels of key proteins. At the functional level, AD patients experience an imbalance in mitochondrial fission and fusion, with a particular increase in excessive mitochondrial fragmentation (increased fission and reduced fusion), which weakens mitochondrial function. Additionally, mitochondrial dynamics within neurons are impaired, particularly in the process of axonal transport. Mitochondria are unable to effectively reach the synaptic terminals, leading to insufficient energy supply to neurons and exacerbating neuronal degeneration and dysfunction. A defect in mitophagy is another significant issue in AD. Under normal conditions, damaged mitochondria are removed through mitophagy to maintain a healthy cellular environment. However, in AD, the mitophagy process is inhibited, preventing damaged mitochondria from being cleared in a timely manner. The accumulation of damaged mitochondria further deteriorates mitochondrial function, creating a feedback loop of degeneration. Moreover, the mitochondrial DNA of AD patients is damaged by abnormal oxidative stress, which not only increases DNA damage but also further impairs mitochondrial function, leading to a continuous destructive cycle. To better understand these mechanisms, future research should focus on regulating mitochondrial function in the context of AD, particularly how mitochondrial regulatory mechanisms during aging influence disease progression. Slight defects in these regulatory mechanisms, determined by genetic background, may serve as a potential trigger for the onset of AD. Furthermore, the dual-membrane structure of mitochondria and the presence of the blood-brain barrier in AD pose significant challenges for drug delivery. Currently, mitochondrial-targeting drug delivery strategies are still in the early stages of development, and TPP derivatives, commonly used as mitochondrial-targeting ligands, have shown side effects, highlighting the need for greater caution in selecting carrier materials. Using greener and safer materials may help reduce toxicity and facilitate clinical translation. By further studying the complexity of these mitochondrial morphological and functional impairments, we can not only better understand the role of mitochondria in AD but also provide new therapeutic targets for all AD patients.

## References

[1] Huang YY Gan YH Yang L Cheng W Yu JT. Depression in Alzheimer’s disease: epidemiology, mechanisms, and treatment. Biol Psychiatry. (2024) 95:992–1005. doi: 10.1016/j.biopsych.2023.10.008 37866486

[2] Guo X Yan L Zhang D Zhao Y. Passive immunotherapy for Alzheimer’s disease. Ageing Res Rev. (2024) 94:102192. doi: 10.1016/j.arr.2024.102192 38219962

[3] Jucker M Walker LC. Alzheimer’s disease: From immunotherapy to immunoprevention. Cell. (2023) 186:4260–70. doi: 10.1016/j.cell.2023.08.021 PMC10578497 37729908

[4] Zhang Y Chen H Li R Sterling K Song W. Amyloid β-based therapy for Alzheimer’s disease: challenges, successes and future. Signal transduction targeted Ther. (2023) 8:248. doi: 10.1038/s41392-023-01484-7 PMC10310781 37386015

[5] Lopez-Lee C Torres ERS Carling G Gan L. Mechanisms of sex differences in Alzheimer’s disease. Neuron. (2024) 112:1208–21. doi: 10.1016/j.neuron.2024.01.024 PMC11076015 38402606

[6] Ma Q Xing C Long W Wang HY Liu Q Wang RF. Impact of microbiota on central nervous system and neurological diseases: the gut-brain axis. J neuroinflammation. (2019) 16:53. doi: 10.1186/s12974-019-1434-3 30823925 PMC6397457

[7] Sun Y Sommerville NR Liu JYH Ngan MP Poon D Ponomarev ED. Intra-gastrointestinal amyloid-β1-42 oligomers perturb enteric function and induce Alzheimer’s disease pathology. J Physiol. (2020) 598:4209–23. doi: 10.1113/jp279919 PMC7586845 32617993

[8] Chen SG Stribinskis V Rane MJ Demuth DR Gozal E Roberts AM. Exposure to the functional bacterial amyloid protein curli enhances alpha-synuclein aggregation in aged fischer 344 rats and Caenorhabditis elegans. Sci Rep. (2016) 6:34477. doi: 10.1038/srep34477 27708338 PMC5052651

[9] Harach T Marungruang N Duthilleul N Cheatham V Mc Coy KD Frisoni G. Reduction of Abeta amyloid pathology in APPPS1 transgenic mice in the absence of gut microbiota. Sci Rep. (2017) 7:41802. doi: 10.1038/srep41802 28176819 PMC5297247

[10] Gowda P Reddy PH Kumar S. Deregulated mitochondrial microRNAs in Alzheimer’s disease: Focus on synapse and mitochondria. Ageing Res Rev. (2022) 73:101529. doi: 10.1016/j.arr.2021.101529 34813976 PMC8692431

[11] Wang W Zhao F Ma X Perry G Zhu X. Mitochondria dysfunction in the pathogenesis of Alzheimer’s disease: recent advances. Mol neurodegeneration. (2020) 15 (35):30. doi: 10.1186/s13024-020-00376-6 PMC7257174 32471464

[12] Mary A Eysert F Checler F Chami M. Mitophagy in Alzheimer’s disease: Molecular defects and therapeutic approaches. Mol Psychiatry. (2023) 28:202–16. doi: 10.1038/s41380-022-01631-6 PMC9812780 35665766

[13] John A Reddy PH. Synaptic basis of Alzheimer’s disease: Focus on synaptic amyloid beta, P-tau and mitochondria. Ageing Res Rev. (2021) 65:101208. doi: 10.1016/j.arr.2020.101208 33157321 PMC7770124

[14] Calvo-Rodriguez M Bacskai BJ. Mitochondria and calcium in Alzheimer’s disease: from cell signaling to neuronal cell death. Trends neurosciences. (2021) 44:136–51. doi: 10.1016/j.tins.2020.10.004 33160650

[15] Maruthiyodan S Mumbrekar KD Guruprasad KP. Involvement of mitochondria in Alzheimer’s disease pathogenesis and their potential as targets for phytotherapeutics. Mitochondrion. (2024) 76:101868. doi: 10.1016/j.mito.2024.101868 38462158

[16] Kadenbach B. Introduction to mitochondrial oxidative phosphorylation. Adv Exp Med Biol. (2012) 748:1–11. doi: 10.1007/978-1-4614-3573-0_1 22729852

[17] Chen H McCaffery JM Chan DC. Mitochondrial fusion protects against neurodegeneration in the cerebellum. Cell. (2007) 130:548–62. doi: 10.1016/j.cell.2007.06.026 17693261

[18] Gibson GE Shi Q. A mitocentric view of Alzheimer’s disease suggests multi-faceted treatments. J Alzheimer’s disease: JAD. (2010) 20 Suppl 2:S591–607. doi: 10.3233/jad-2010-100336 PMC3085842 20463407

[19] Guo L Tian J Du H. Mitochondrial dysfunction and synaptic transmission failure in Alzheimer’s disease. J Alzheimer’s disease: JAD. (2017) 57:1071–86. doi: 10.3233/jad-160702 PMC5605817 27662318

[20] Liang WS Reiman EM Valla J Dunckley T Beach TG Grover A. Alzheimer’s disease is associated with reduced expression of energy metabolism genes in posterior cingulate neurons. Proc Natl Acad Sci U S A. (2008) 105:4441–6. doi: 10.1073/pnas.0709259105 PMC2393743 18332434

[21] Brooks WM Lynch PJ Ingle CC Hatton A Emson PC Faull RL. Gene expression profiles of metabolic enzyme transcripts in Alzheimer’s disease. Brain Res. (2007) 1127:127–35. doi: 10.1016/j.brainres.2006.09.106 17109828

[22] Adav SS Park JE Sze SK. Quantitative profiling brain proteomes revealed mitochondrial dysfunction in Alzheimer’s disease. Mol brain. (2019) 12:8. doi: 10.1186/s13041-019-0430-y 30691479 PMC6350377

[23] Garcia-Casas P Rossini M Filadi R Pizzo P. Mitochondrial Ca(2+) signaling and Alzheimer’s disease: Too much or too little? Cell calcium. (2023) 113:102757. doi: 10.1016/j.ceca.2023.102757 37192560

[24] Hadi F Mortaja M Hadi Z. Calcium (Ca(2+)) hemostasis, mitochondria, autophagy, and mitophagy contribute to Alzheimer’s disease as early moderators. Cell Biochem Funct. (2024) 42:e4085. doi: 10.1002/cbf.4085 38951992

[25] Trushina E Trushin S Hasan MF. Mitochondrial complex I as a therapeutic target for Alzheimer’s disease. Acta Pharm Sin B. (2022) 12:483–95. doi: 10.1016/j.apsb.2021.11.003 PMC8897152 35256930

[26] Cai Q Tammineni P. Alterations in mitochondrial quality control in Alzheimer’s disease. Front Cell Neurosci. (2016) 10:24. doi: 10.3389/fncel.2016.00024 26903809 PMC4746252

[27] Zhu X Castellani RJ Moreira PI Aliev G Shenk JC Siedlak SL. Hydroxynonenal-generated crosslinking fluorophore accumulation in Alzheimer disease reveals a dichotomy of protein turnover. Free Radical Biol Med. (2012) 52:699–704. doi: 10.1016/j.freeradbiomed.2011.11.004 22137893 PMC3268699

[28] Misrani A Tabassum S Yang L. Mitochondrial dysfunction and oxidative stress in Alzheimer’s disease. Front Aging Neurosci. (2021) 13:617588. doi: 10.3389/fnagi.2021.617588 33679375 PMC7930231

[29] Du F Yu Q Kanaan NM Yan SS. Mitochondrial oxidative stress contributes to the pathological aggregation and accumulation of tau oligomers in Alzheimer’s disease. Hum Mol Genet. (2022) 31:2498–507. doi: 10.1093/hmg/ddab363 PMC9396941 35165721

[30] Wang P Cui Y Ren Q Yan B Zhao Y Yu P. Mitochondrial ferritin attenuates cerebral ischaemia/reperfusion injury by inhibiting ferroptosis. Cell Death disease. (2021) 12:447. doi: 10.1038/s41419-021-03725-5 33953171 PMC8099895

[31] Wu Q Wu WS Su L Zheng X Wu WY Santambrogio P. Mitochondrial ferritin is a hypoxia-inducible factor 1α-inducible gene that protects from hypoxia-induced cell death in brain. Antioxidants Redox Signaling. (2019) 30:198–212. doi: 10.1089/ars.2017.7063 29402144

[32] Cheng J Dong Y Ma J Pan R Liao Y Kong X. Microglial Calhm2 regulates neuroinflammation and contributes to Alzheimer’s disease pathology. Sci Adv. (2021) 7:eabe3600. doi: 10.1126/sciadv.abe3600 PMC8386937 34433553

[33] Ionescu-Tucker A Cotman CW. Emerging roles of oxidative stress in brain aging and Alzheimer’s disease. Neurobiol aging. (2021) 107:86–95. doi: 10.1016/j.neurobiolaging.2021.07.014 34416493

[34] Garg G Singh S Singh AK Rizvi SI. N-acetyl-l-cysteine attenuates oxidative damage and neurodegeneration in rat brain during aging. Can J Physiol Pharmacol. (2018) 96:1189–96. doi: 10.1139/cjpp-2018-0209 30107137

[35] Liu S Fu S Wang G Cao Y Li L Li X. Glycerol-3-phosphate biosynthesis regenerates cytosolic NAD(+) to alleviate mitochondrial disease. Cell Metab. (2021) 33:1974–87. doi: 10.1016/j.cmet.2021.06.013 34270929

[36] Sayehmiri F Motamedi F Batool Z Naderi N Shaerzadeh F Zoghi A. Mitochondrial plasticity and synaptic plasticity crosstalk; in health and Alzheimer’s disease. CNS Neurosci Ther. (2024) 30:e14897. doi: 10.1111/cns.14897 39097920 PMC11298206

[37] Pszczołowska M Walczak K Miśków W Mroziak M Chojdak-Łukasiewicz J Leszek J. Mitochondrial disorders leading to Alzheimer’s disease-perspectives of diagnosis and treatment. GeroScience. (2024) 46:2977–88. doi: 10.1007/s11357-024-01118-y PMC11009177 38457008

[38] Swerdlow RH. Mitochondria and mitochondrial cascades in Alzheimer’s disease. J Alzheimer’s disease: JAD. (2018) 62:1403–16. doi: 10.3233/jad-170585 PMC5869994 29036828

[39] Nasb M Tao W Chen N. Alzheimer’s disease puzzle: delving into pathogenesis hypotheses. Aging disease. (2024) 15:43–73. doi: 10.14336/ad.2023.0608 37450931 PMC10796101

[40] Yoo SM Park J Kim SH Jung YK. Emerging perspectives on mitochondrial dysfunction and inflammation in Alzheimer’s disease. BMB Rep. (2020) 53:35–46. doi: 10.5483/BMBRep.2020.53.1.274 31818363 PMC6999830

[41] Bano D Ehninger D Bagetta G. Decoding metabolic signatures in Alzheimer’s disease: a mitochondrial perspective. Cell Death discov. (2023) 9:432. doi: 10.1038/s41420-023-01732-3 38040687 PMC10692234

[42] Wang X Su B Siedlak SL Moreira PI Fujioka H Wang Y. Amyloid-beta overproduction causes abnormal mitochondrial dynamics via differential modulation of mitochondrial fission/fusion proteins. Proc Natl Acad Sci U S A. (2008) 105:19318–23. doi: 10.1073/pnas.0804871105 PMC2614759 19050078

[43] Wang X Su B Lee HG Li X Perry G Smith MA. Impaired balance of mitochondrial fission and fusion in Alzheimer’s disease. J Neurosci. (2009) 29:9090–103. doi: 10.1523/jneurosci.1357-09.2009 PMC2735241 19605646

[44] Shields LY Kim H Zhu L Haddad D Berthet A Pathak D. Dynamin-related protein 1 is required for normal mitochondrial bioenergetic and synaptic function in CA1 hippocampal neurons. Cell Death disease. (2015) 6:e1725. doi: 10.1038/cddis.2015.94 25880092 PMC4650558

[45] Manczak M Kandimalla R Fry D Sesaki H Reddy PH. Protective effects of reduced dynamin-related protein 1 against amyloid beta-induced mitochondrial dysfunction and synaptic damage in Alzheimer’s disease. Hum Mol Genet. (2016) 25:5148–66. doi: 10.1093/hmg/ddw330 PMC6078633 27677309

[46] Kleele T Rey T Winter J Zaganelli S Mahecic D Perreten Lambert H. Distinct fission signatures predict mitochondrial degradation or biogenesis. Nature. (2021) 593:435–9. doi: 10.1038/s41586-021-03510-6 33953403

[47] Xu LL Shen Y Wang X Wei LF Wang P Yang H. Mitochondrial dynamics changes with age in an APPsw/PS1dE9 mouse model of Alzheimer’s disease. Neuroreport. (2017) 28:222–8. doi: 10.1097/wnr.0000000000000739 PMC5321113 28118288

[48] Liu W Acín-Peréz R Geghman KD Manfredi G Lu B Li C. Pink1 regulates the oxidative phosphorylation machinery via mitochondrial fission. Proc Natl Acad Sci U S A. (2011) 108:12920–4. doi: 10.1073/pnas.1107332108 PMC3150934 21768365

[49] Cho DH Nakamura T Fang J Cieplak P Godzik A Gu Z. S-nitrosylation of Drp1 mediates beta-amyloid-related mitochondrial fission and neuronal injury. Sci (New York NY). (2009) 324:102–5. doi: 10.1126/science.1171091 PMC2823371 19342591

[50] D’Souza AR Minczuk M. Mitochondrial transcription and translation: overview. Essays Biochem. (2018) 62:309–20. doi: 10.1042/ebc20170102 PMC6056719 30030363

[51] Song M Ye L Yan Y Li X Han X Hu S. Mitochondrial diseases and mtDNA editing. Genes diseases. (2024) 11:101057. doi: 10.1016/j.gendis.2023.06.026 38292200 PMC10825299

[52] Zong Y Li H Liao P Chen L Pan Y Zheng Y. Mitochondrial dysfunction: mechanisms and advances in therapy. Signal transduction targeted Ther. (2024) 9:124. doi: 10.1038/s41392-024-01839-8 PMC11094169 38744846

[53] Trifunovic A Wredenberg A Falkenberg M Spelbrink JN Rovio AT Bruder CE. Premature ageing in mice expressing defective mitochondrial DNA polymerase. Nature. (2004) 429:417–23. doi: 10.1038/nature02517 15164064

[54] Pienaar IS Howell N Elson JL. MutPred mutational load analysis shows mildly deleterious mitochondrial DNA variants are not more prevalent in Alzheimer’s patients, but may be under-represented in healthy older individuals. Mitochondrion. (2017) 34:141–6. doi: 10.1016/j.mito.2017.04.002 28396254

[55] Zhang H Zhu Y Suehiro Y Mitani S Xue D. AMPK-FOXO-IP3R signaling pathway mediates neurological and developmental defects caused by mitochondrial DNA mutations. Proc Natl Acad Sci U S A. (2023) 120:e2302490120. doi: 10.1073/pnas.2302490120 37639584 PMC10483642

[56] Chen Y Liu C Parker WD Chen H Beach TG Liu X. Mitochondrial DNA rearrangement spectrum in brain tissue of Alzheimer’s disease: analysis of 13 cases. PLoS One. (2016) 11:e0154582. doi: 10.1371/journal.pone.0154582 27299301 PMC4907522

[57] Soltys DT Pereira CPM Rowies FT Farfel JM Grinberg LT Suemoto CK. Lower mitochondrial DNA content but not increased mutagenesis associates with decreased base excision repair activity in brains of AD subjects. Neurobiol aging. (2019) 73:161–70. doi: 10.1016/j.neurobiolaging.2018.09.015 30359878

[58] Coskun PE Beal MF Wallace DC. Alzheimer’s brains harbor somatic mtDNA control-region mutations that suppress mitochondrial transcription and replication. Proc Natl Acad Sci U S A. (2004) 101:10726–31. doi: 10.1073/pnas.0403649101 PMC490002 15247418

[59] Wang J Xiong S Xie C Markesbery WR Lovell MA. Increased oxidative damage in nuclear and mitochondrial DNA in Alzheimer’s disease. J neurochemistry. (2005) 93:953–62. doi: 10.1111/j.1471-4159.2005.03053.x 15857398

[60] Canugovi C Shamanna RA Croteau DL Bohr VA. Base excision DNA repair levels in mitochondrial lysates of Alzheimer’s disease. Neurobiol aging. (2014) 35:1293–300. doi: 10.1016/j.neurobiolaging.2014.01.004 PMC5576885 24485507

[61] Shao C Xiong S Li GM Gu L Mao G Markesbery WR. Altered 8-oxoguanine glycosylase in mild cognitive impairment and late-stage Alzheimer’s disease brain. Free Radical Biol Med. (2008) 45:813–9. doi: 10.1016/j.freeradbiomed.2008.06.003 PMC2745061 18598755

[62] Hoekstra JG Hipp MJ Montine TJ Kennedy SR. Mitochondrial DNA mutations increase in early stage Alzheimer disease and are inconsistent with oxidative damage. Ann neurol. (2016) 80:301–6. doi: 10.1002/ana.24709 PMC4982791 27315116

[63] Blanch M Mosquera JL Ansoleaga B Ferrer I BarraChina M. Altered mitochondrial DNA methylation pattern in Alzheimer disease-related pathology and in Parkinson disease. Am J pathol. (2016) 186:385–97. doi: 10.1016/j.ajpath.2015.10.004 26776077

[64] Stoccoro A Siciliano G Migliore L Coppedè F. Decreased methylation of the mitochondrial D-loop region in late-onset Alzheimer’s disease. J Alzheimer’s disease: JAD. (2017) 59:559–64. doi: 10.3233/jad-170139 28655136

[65] Pradeepkiran JA Reddy PH. Defective mitophagy in Alzheimer’s disease. Ageing Res Rev. (2020) 64:101191. doi: 10.1016/j.arr.2020.101191 33022416 PMC7710581

[66] Zeng K Yu X Mahaman YAR Wang JZ Liu R Li Y. Defective mitophagy and the etiopathogenesis of Alzheimer’s disease. Trans neurodegeneration. (2022) 11:32. doi: 10.1186/s40035-022-00305-1 PMC9164340 35655270

[67] Harper JW Ordureau A Heo JM. Building and decoding ubiquitin chains for mitophagy. Nat Rev Mol Cell Biol. (2018) 19:93–108. doi: 10.1038/nrm.2017.129 29358684

[68] Sekine S Youle RJ. PINK1 import regulation; a fine system to convey mitochondrial stress to the cytosol. BMC Biol. (2018) 16:2. doi: 10.1186/s12915-017-0470-7 29325568 PMC5795276

[69] Pickles S Vigié P Youle RJ. Mitophagy and quality control mechanisms in mitochondrial maintenance. Curr biol: CB. (2018) 28:R170–r185. doi: 10.1016/j.cub.2018.01.004 29462587 PMC7255410

[70] Chen M Chen Z Wang Y Tan Z Zhu C Li Y. Mitophagy receptor FUNDC1 regulates mitochondrial dynamics and mitophagy. Autophagy. (2016) 12:689–702. doi: 10.1080/15548627.2016.1151580 27050458 PMC4836026

[71] Liu L Feng D Chen G Chen M Zheng Q Song P. Mitochondrial outer-membrane protein FUNDC1 mediates hypoxia-induced mitophagy in mammalian cells. Nat Cell Biol. (2012) 14:177–85. doi: 10.1038/ncb2422 22267086

[72] Li J Li M Ge Y Chen J Ma J Wang C. [amp]]beta;-amyloid protein induces mitophagy-dependent ferroptosis through the CD36/PINK/PARKIN pathway leading to blood-brain barrier destruction in Alzheimer’s disease. Cell biosci. (2022) 12:69. doi: 10.1186/s13578-022-00807-5 35619150 PMC9134700

[73] Chen J He HJ Ye Q Feng F Wang WW Gu Y. Defective autophagy and mitophagy in Alzheimer’s disease: mechanisms and translational implications. Mol neurobiol. (2021) 58:5289–302. doi: 10.1007/s12035-021-02487-7 34279771

[74] Cao Y Liu B Xu W Wang L Shi F Li N. Inhibition of mTORC1 improves STZ-induced AD-like impairments in mice. Brain Res bulletin. (2020) 162:166–79. doi: 10.1016/j.brainresbull.2020.06.002 32599128

[75] Wang X Jia J. Magnolol improves Alzheimer’s disease-like pathologies and cognitive decline by promoting autophagy through activation of the AMPK/mTOR/ULK1 pathway. Biomed pharmacother = Biomed pharmacotherapie. (2023) 161:114473. doi: 10.1016/j.biopha.2023.114473 36889111

[76] Pan XJ Misrani A Tabassum S Yang L. Mitophagy pathways and Alzheimer’s disease: From pathogenesis to treatment. Mitochondrion. (2021) 59:37–47. doi: 10.1016/j.mito.2021.04.007 33872797

[77] Fang EF Hou Y Palikaras K Adriaanse BA Kerr JS Yang B. Mitophagy inhibits amyloid-β and tau pathology and reverses cognitive deficits in models of Alzheimer’s disease. Nat Neurosci. (2019) 22:401–12. doi: 10.1038/s41593-018-0332-9 PMC6693625 30742114

[78] Torres AK Rivera BI Polanco CM Jara C Tapia-Rojas C. Phosphorylated tau as a toxic agent in synaptic mitochondria: implications in aging and Alzheimer’s disease. Neural regeneration Res. (2022) 17:1645–51. doi: 10.4103/1673-5374.332125 PMC8820692 35017410

[79] Limanaqi F Biagioni F Gambardella S Familiari P Frati A Fornai F. Promiscuous roles of autophagy and proteasome in neurodegenerative proteinopathies. Int J Mol Sci. (2020) 21 (8):3028. doi: 10.3390/ijms21083028 PMC7215558 32344772

[80] Eckert A Schulz KL Rhein V Götz J. Convergence of amyloid-beta and tau pathologies on mitochondria *in vivo* . Mol Neurobiol. (2010) 41:107–14. doi: 10.1007/s12035-010-8109-5 PMC2876263 20217279

[81] Sun K Jing X Guo J Yao X Guo F. Mitophagy in degenerative joint diseases. Autophagy. (2021) 17:2082–92. doi: 10.1080/15548627.2020.1822097 PMC8496714 32967533

[82] Ke J Tian Q Xu Q Fu Z Fu Q. Mitochondrial dysfunction: A potential target for Alzheimer’s disease intervention and treatment. Drug Discovery Today. (2021) 26:1991–2002. doi: 10.1016/j.drudis.2021.04.025 33962036

[83] Vidoni ED Choi IY Lee P Reed G Zhang N Pleen J. Safety and target engagement profile of two oxaloacetate doses in Alzheimer’s patients. Alzheimer’s dementia: J Alzheimer’s Assoc. (2021) 17:7–17. doi: 10.1002/alz.12156 PMC8084114 32715609

[84] Li H Zhang D Wang X Wang S Xiao M. Protective effect of glutamic-oxaloacetic transaminase on hippocampal neurons in Alzheimer’s disease using model mice. Neurosci letters. (2023) 803:137194. doi: 10.1016/j.neulet.2023.137194 36931592

[85] Panes J Nguyen TKO Gao H Christensen TA Stojakovic A Trushin S. Trushina E. Partial inhibition of complex I restores mitochondrial morphology and mitochondria-ER communication in hippocampus of APP/PS1 mice. Cells. (2023) 12 (8):1111. doi: 10.3390/cells12081111 PMC10137328 37190020

[86] George AJ Gordon L Beissbarth T Koukoulas I Holsinger RM Perreau V. A serial analysis of gene expression profile of the Alzheimer’s disease Tg2576 mouse model. Neurotoxicity Res. (2010) 17:360–79. doi: 10.1007/s12640-009-9112-3 19760337

[87] Kumar A Singh A. A review on mitochondrial restorative mechanism of antioxidants in Alzheimer’s disease and other neurological conditions. Front Pharmacol. (2015) 6:206. doi: 10.3389/fphar.2015.00206 26441662 PMC4585235

[88] Cowan K Anichtchik O Luo S. Mitochondrial integrity in neurodegeneration. CNS Neurosci Ther. (2019) 25:825–36. doi: 10.1111/cns.13105 PMC6566061 30746905

[89] McManus MJ Murphy MP Franklin JL. The mitochondria-targeted antioxidant MitoQ prevents loss of spatial memory retention and early neuropathology in a transgenic mouse model of Alzheimer’s disease. J Neurosci. (2011) 31:15703–15. doi: 10.1523/jneurosci.0552-11.2011 PMC3334845 22049413

[90] Young ML Franklin JL. The mitochondria-targeted antioxidant MitoQ inhibits memory loss, neuropathology, and extends lifespan in aged 3xTg-AD mice. Mol Cell neurosciences. (2019) 101:103409. doi: 10.1016/j.mcn.2019.103409 PMC7359863 31521745

[91] Ding XW Robinson M Li R Aldhowayan H Geetha T Babu JR. Mitochondrial dysfunction and beneficial effects of mitochondria-targeted small peptide SS-31 in Diabetes Mellitus and Alzheimer’s disease. Pharmacol Res. (2021) 171:105783. doi: 10.1016/j.phrs.2021.105783 34302976

[92] Mota IFL de Lima LS Santana BM Gobbo GAM Bicca J Azevedo JRM. Alzheimer’s disease: innovative therapeutic approaches based on peptides and nanoparticles. Neuroscientist: Rev J bringing neurobiol Neurol Psychiatry. (2023) 29:78–96. doi: 10.1177/10738584211016409 34018874

[93] Han Y Gao C Wang H Sun J Liang M Feng Y. Macrophage membrane-coated nanocarriers Co-Modified by RVG29 and TPP improve brain neuronal mitochondria-targeting and therapeutic efficacy in Alzheimer’s disease mice. Bioactive materials. (2021) 6:529–42. doi: 10.1016/j.bioactmat.2020.08.017 PMC7492821 32995678

[94] Buchke S Sharma M Bora A Relekar M Bhanu P Kumar J. Mitochondria-targeted, nanoparticle-based drug-delivery systems: therapeutics for mitochondrial disorders. Life (Basel). (2022) 12 (5):657. doi: 10.3390/life12050657 PMC9144057 35629325

[95] Liu Y Shen Y. Applications of nanoparticles in Alzheimer’s disease. J Alzheimer’s disease: JAD. (2023) 96:459–71. doi: 10.3233/jad-230098 37807779

[96] Zhong G Long H Zhou T Liu Y Zhao J Han J. Blood-brain barrier Permeable nanoparticles for Alzheimer’s disease treatment by selective mitophagy of microglia. Biomaterials. (2022) 288:121690. doi: 10.1016/j.biomaterials.2022.121690 35965114

[97] Cores Á Carmona-Zafra N Clerigué J Villacampa M Menéndez JC. Quinones as neuroprotective agents. Antioxidants (Basel). (2023) 12 (7):1464. doi: 10.3390/antiox12071464 PMC10376830 37508002

[98] Yang S Wang L Xie Z Zeng Y Xiong Q Pei T. The combination of salidroside and hedysari radix polysaccharide inhibits mitochondrial damage and apoptosis via the PKC/ERK pathway. Evidence-Based complementary Altern medicine: eCAM. (2022) 2022:9475703. doi: 10.1155/2022/9475703 PMC9252633 35795284

[99] Liu X Song L Yu J Huang F Li Y Ma C. Mdivi-1: a promising drug and its underlying mechanisms in the treatment of neurodegenerative diseases. Histol histopathol. (2022) 37:505–12. doi: 10.14670/hh-18-443 35199329

[100] Oliver D Reddy PH. Dynamics of dynamin-related protein 1 in Alzheimer’s disease and other neurodegenerative diseases. Cells. (2019) 8. doi: 10.3390/cells8090961 PMC6769467 31450774

[101] Austad SN Ballinger S Buford TW Carter CS Smith DL Jr. Darley-Usmar V. Targeting whole body metabolism and mitochondrial bioenergetics in the drug development for Alzheimer’s disease. Acta Pharm Sin B. (2022) 12:511–31. doi: 10.1016/j.apsb.2021.06.014 PMC8897048 35256932

[102] Payne A Nahashon S Taka E Adinew GM Soliman KFA. Epigallocatechin-3-gallate (EGCG): new therapeutic perspectives for neuroprotection, aging, and neuroinflammation for the modern age. Biomolecules. (2022) 12 (3):371. doi: 10.3390/biom12030371 PMC8945730 35327563

[103] Mohamad Ishak NS Kikuchi M Ikemoto K. Dietary pyrroloquinoline quinone hinders aging progression in male mice and D-galactose-induced cells. Front aging. (2024) 5:1351860. doi: 10.3389/fragi.2024.1351860 38487591 PMC10938241

[104] Yang AJT Bagit A MacPherson REK. Resveratrol, metabolic dysregulation, and Alzheimer’s disease: considerations for neurogenerative disease. Int J Mol Sci. (2021) 22 (9):4628. doi: 10.3390/ijms22094628 PMC8125227 33924876

[105] Wang Y Hu H Liu X Guo X. Hypoglycemic medicines in the treatment of Alzheimer’s disease: Pathophysiological links between AD and glucose metabolism. Front Pharmacol. (2023) 14:1138499. doi: 10.3389/fphar.2023.1138499 36909158 PMC9995522

[106] Fairley LH Lejri I Grimm A Eckert A. Spermidine rescues bioenergetic and mitophagy deficits induced by disease-associated tau protein. Int J Mol Sci. (2023) 24 (6):5297. doi: 10.3390/ijms24065297 PMC10049002 36982371

